# Acquired Factor VIII Deficiency Presenting as Gross Hematuria in a Hispanic, Pregnant Patient with Previously Undiagnosed Connective Tissue Disease

**DOI:** 10.1155/2021/3666270

**Published:** 2021-11-17

**Authors:** Christine Loftis, Emilia C. Dulgheru, Rosa White

**Affiliations:** ^1^University of Texas at Rio Grande Valley, Internal Medicine Department- Doctor Hospital at Renaissance, 5423 S McColl Rd, Edinburg, TX 78539, USA; ^2^Rheumatology Institute-Doctor Hospital at Renaissance, Edinburg, TX 78539, USA

## Abstract

Acquired factor VIII deficiency is a bleeding disorder caused by the presence of autoantibodies against clotting factor VIII. We report a case of a 24-year-old pregnant woman who presented with gross hematuria secondary to acquired factor VIII deficiency in the presence of a previously undiagnosed connective tissue disease. This article includes a literature review of pregnancy-related cases of acquired factor VIII deficiency. We also reviewed various therapeutic approaches for the management of the acquired factor inhibitor which include achieving hemostasis and elimination of the inhibitor via immunosuppressive agents. This case report describes the rare presentation of acquired factor VIII deficiency related to pregnancy and highlights the importance of considering a factor VIII inhibitor in the differential diagnosis of patients who present with bleeding and prolonged PTT during the peripartum and postpartum periods.

## 1. Introduction

Acquired factor VIII deficiency is a rare bleeding diathesis caused by the development of autoantibodies against clotting factor VIII [1]. The annual incidence of the disorder is between 1.3 and 1.5 per million [1]. Apart from reproductive-aged women during pregnancy and postpartum periods, acquired factor VIII deficiency typically occurs after the fifth decade of life [2]. Patients affected with acquired factor VIII deficiency present with severe or life-threatening bleeding episodes in the absence of previous bleeding predisposition. Bleeding severity varies ranging from simple dermatological manifestations such as petechiae and ecchymoses to internal bleeding [3] Acquired factor VIII deficiency has been associated with several underlying conditions such as malignancy, immunological disorders, perinatal period, and various medications. Although the association with underlying disorders has been reported, most of the cases remain idiopathic.

## 2. Case Presentation

A 24-year-old Hispanic pregnant woman, gravida 2 para 1, was admitted for gross hematuria without precipitating factors at 29 weeks of gestation. The patient reported that this was the first episode of hematuria and had no prior history of bleeding during her previous pregnancy or otherwise. The patient's review of systems was significant for an erythematous, maculopapular, nonpruritic rash, occurring intermittently on the lower extremities since age 13. The patient had never been given a formal diagnosis but noted that the rash also occurred during her first pregnancy, resolved spontaneously, and recurred about 2 weeks prior to the current admission. The patient denied family history of hemophilia, von Willebrand disease, or autoimmune conditions such as rheumatoid arthritis, systemic lupus erythematosus, scleroderma, or Hashimoto's thyroiditis. The patient endorsed episodes of photosensitivity but denied constitutional symptoms, arthralgia, oral ulcers, myalgia, pleuritic chest pain, xerostomia, keratoconjunctivitis sicca, or any other significant symptoms. On physical examination, the patient had bilateral upper extremity and lower back large ecchymoses, a palpable purpura on the anterior aspect of the right thigh, bilateral knees, and hips, and postinflammatory hyperpigmentation on the distal aspect of lower extremities. Laboratory tests (Table 1) showed a prolonged PTT of 92.1 s (RI: 24.3–36.7 s). Subsequent workup revealed negative antiphospholipid panel including negative pus anticoagulant, cardiolipin antibodies, and beta-2 glycoprotein 1 antibodies. The von Willebrand factor was present in adequate amounts. There was a significantly decreased factor VIII level of <1% (RI: 50–180%), and PTT failed to correct with the addition of fresh plasma (mixing studies) which suggested the presence of an inhibitor. Bethesda assay was positive at 74.9 U (RI: 0.6 U) which confirmed a high titer factor VIII inhibitor. Further workup revealed a positive antinuclear antibody with high titer by ELISA and positive SS-A and SS-B antibodies. Urinalysis showed proteinuria of 858 mg/24 h. Double-stranded DNA and anti-Smith antibodies were negative. Complement C3 and C4 were present in normal amounts. The patient was diagnosed with acquired factor VIII deficiency and presumed systemic lupus erythematosus (SLE). She was initially treated with DDAVP 30 mcg intranasally along with prednisone 45 mg BID (1 mg/kg a day in divided doses) while in our facility. The patient was transferred to a tertiary center for a higher level of care where she received IVIG (intravenous immune globulin) 1 gm/kg for 2 days along with porcine factor VIII. She was discharged from the hospital on a prednisone taper. The patient was followed closely by a maternal-fetal medicine specialist and a hemophilia specialist. The patient later delivered a healthy baby girl at 36 weeks via vaginal delivery without complications. At the three-year follow-up, the patient remains in remission, and her daughter has been without any cardiac problems or bleeding diathesis. She was advised against future pregnancy.

## 3. Discussion

Acquired factor VIII deficiency associated with pregnancy most commonly occurs during the peripartum and postpartum period and rarely occurs before pregnancy. The cause and timing remain unclear, but many studies speculate that it is due to the complex immunological changes that occur during pregnancy. Some sources have theorized that the development of the inhibitor is due to sensitization of the mother's immune system to fetal factor VIII during previous pregnancies; however, some patients who acquire an inhibitor to factor VIII develop the inhibitor in the first pregnancy (Table 2). The inhibitor autoantibody that is produced in patients with acquired factor VIII deficiency attaches to a specific subunit of factor VIII, the C2 domain, which results in diminished procoagulant activity and subsequent bleeding [16]. This is like the formation of neutralizing antibodies against factor VIII in 25% of patients with hemophilia A who are transfused with plasma-derived or recombinant factor VIII. In the rare event of clinical manifestations during pregnancy, such as in our patient, the presence of factor VIII autoantibodies also puts the fetus at risk of bleeding due to the maternofetal transplacental passage of IgG antibodies. To date, there are less than fifty reported cases of the maternal transplacental passage of the inhibitor resulting in acquired factor VIII deficiency in the fetus. Although rare, it is important to counsel the patient on the possibility of these risks [17]. There is no current accurate way to determine the severity of bleeding in patients with factor inhibitors based solely on titer levels. Studies have shown that inhibitors to factor VIII are cleared in a nonlinear manner, thus resulting in an underestimation of bleeding risk in some patients [18].

Acquired factor VIII deficiency occurs most commonly in primigravid women at 2-3 months postpartum. These patients typically present with soft tissue bleeding; however, vaginal bleeding, hemarthrosis, and hematuria have been the presenting symptoms in 13–18% of the reported cases [3]. Although our patient was gravida 2, she delivered her first child 2.5 months prior to the conception that resulted in her current pregnancy. As the immunology of pregnancy poses a conundrum, the presence of an untreated autoimmune condition could have potentially interfered in the dynamic of antibody formation and clearance in our patient.

The isolated elevated PTT that failed to correct with the mixing study in the context of bleeding is suggestive of the inhibitor rather than lupus anticoagulant with latter triggering clotting events. Laboratory testing was negative for the lupus anticoagulant, but factor VIII activity was significantly decreased at less than 1%. Bethesda assay was positive at 74.9 U confirming the presence of an inhibitor to factor VIII. A unique aspect of our case was the importance of recognizing the presentation of a previously undiagnosed connective tissue disorder.

The patient reported intermittent erythematous rash reminding of purpura as well as photosensitivity since age 13, without any formal evaluation or diagnosis. Additional testing during current pregnancy showed the presence of positive ANA, SS-A, and SS-B antibodies in addition to proteinuria, suggesting a diagnosis of SLE [19].

In addition to potential bleeding occurrence in the fetus, the possible passive transplacental transfer of maternal anti-SS-A and SS-B IgG antibodies incurs the risk of neonatal lupus [20]. These presented a double risk for our patient that could have potentially increased morbidity.

The phenomenon that resulted in developing an inhibitor to factor VIII could represent a concerted occurrence of undiagnosed SLE as an autoimmune situation and the short interpregnancy interval between the patient's prior and current pregnancy and the hormonal changes associated. As the development of factor VIII inhibitors typically occurs in the 2-3 postpartum period, the patient became pregnant around the same interval, and the amplification of the process leading to high-titer pathogenic antibodies could have been caused by immunological processes of both undiagnosed SLE and occurrence of subsequent pregnancy. Flares of SLE with the characteristic antibody production that characterize the disease occur frequently in the postpartum period.

The immunology of pregnancy in SLE poses a paradoxical juncture of immune tolerance required to insure the viability of semiallogenic graft and the disturbance of the tolerance characteristic of SLE. The most important immune alteration is related to the function of Treg cells. The number or the functionality of Treg cells is diminished in a disease characterized by loss of immune tolerance such as SLE, whereas the immune tolerance of the fetus is insured by Treg in a normal pregnancy. Tregs' presence is correlated with the pregnancy outcome. Lower Tregs are associated with pregnancy complications. Moreover, the immunological processes of pregnancy are complicated by the confrontation of two separate immune systems—the maternal immune system and the fetal-placental immune system [21].

## 4. Literature Review

Our literature review identified fifteen cases of acquired factor VIII deficiency associated with pregnancy with patient's ages ranging between 18 and 37 years. The literature indicates that most cases of acquired factor VIII inhibitors develop after the first pregnancy and typically between 2 and 3 months postpartum. Based on our literature review, we did note that several of the patients developed acquired factor VIII inhibitors after delivering their first child; however, most patients developed postpartum hemorrhage within days of delivery [5, 7–10]. There were other primigravid cases where the patient developed acquired factor VIII inhibitors, but symptoms did not manifest until 2–9 months postpartum [4, 12, 15, 22]. Still, there were cases of acquired factor VIII deficiency in women after their second pregnancy [14, 17] and third pregnancy [4]. There did not seem to be any correlation with the gravid status of the patient and clinical outcomes. Of the fifteen cases reviewed, two patients died from complications related directly to the acquired factor deficiency in the setting of hemorrhagic shock [9] and indirectly secondary to sepsis in the setting of immunosuppression for eradication therapy [20]. These two women were aged 19 and 36, respectively, without other comorbid conditions and had initial inhibitor levels within the 50–60 BU range. An important aspect of each of the aforementioned cases is that each patient had significant vaginal bleeding on presentation. In the former case, the patient presented with a large vaginal hematoma with intra-abdominal bleeding, and the latter developed uncontrolled vaginal bleeding for which she delayed treatment for due to travel between the United States and Mexico. It is unclear whether the prompt intervention could have improved outcomes in these patients, but it is important to consider the acquired factor VIII inhibitor as a differential in a patient with persistent vaginal bleeding during or after pregnancy. Of note, there were three patients (including our case) who developed an acquired factor deficiency prior to delivery [6, 11]. As mentioned previously, our patient developed the inhibitor during her second pregnancy. However, she had a short interval between her first and second pregnancy. One of the cases of acquired factor deficiency prior to delivery was in a patient with an underlying diagnosis of SLE [11]. During our literature search, we did not identify other cases reported of concomitant acquired factor deficiency in pregnant patients with underlying connective tissue diseases. As noted, our patient had an eventful delivery; however, the aforementioned patient had to terminate the pregnancy due to retroperitoneal bleeding which increased the risk for mortality for both patient and fetus.

Regarding the titer level and severity of bleeding, there appeared to be a higher requirement for blood products, worsening of bleeding diathesis, and, in some cases, worse outcomes with a Bethesda titer >100 BU [4, 8, 9, 11]. In one of the cases, the patient inhibitor level increased from the initial level of 64 BU to a level as high as 132 BU. As previously mentioned, this patient died due to hemorrhagic shock on postpartum day 8 [9]. Likewise, the case of acquired factor VIII concomitantly with SLE resulted in a massive retroperitoneal bleed. This patient had an initial titer of 614 BU; however, the patient was able to successfully clear the inhibitor although she did have to terminate pregnancy due to the risk of worsening complications if she continued to term [11]. Moreover, Michiels et al. described a case of a young woman who delivered a stillborn and subsequently developed postpartum hemorrhage with titers as high as 625 BU [4]. This patient did not receive remission at follow-up visits 24 years later despite immunosuppressive therapy. Lastly, Kotani et al. described a case of postpartum hemorrhage with an initial titer level of 458 BU. This patient along with her neonate developed complications; however, after the patient received several blood products including nineteen packed red blood cells, fifty-one units of fresh frozen plasma, 4.8 mg of factor VIIa, and pulse steroid therapy, the patient developed remission [8]. Previous studies note that severity of bleeding does not correlate with the titer level; however, our literature review shows that the severity of bleeding is at least correlated with the titer level of the inhibitor level which reaches as high as 100 BU.

Remission was achieved in most patients by a two-step process aimed at controlling the bleeding and eliminating the inhibitor. Our literature review showed that several patients were treated with some combinations of either desmopressin, factor eight inhibitor bypassing activity (FEIBA), recombinant human factor VIIa, or recombinant porcine factor VIII concentrate, tranexamic acid, or some other blood products including packed red blood cells or fresh frozen plasma. Several agents were used for eradication of the inhibitor including prednisone, methylprednisolone, cyclophosphamide, rituximab, and azathioprine. The European Acquired Hemophilia Registry (EACH 2) reported 42 documented cases of acquired factor VIII deficiency associated with the peripartum period out of the total 501 documented cases. Of the 42 cases, 74% of patients acquired complete remission with first-line immunosuppressive treatment with all women being alive at the last follow-up visit. To date, there has not been a registry developed in the United States.

## 5. Learning Points

Acquired factor VIII hemophilia secondary to the coagulation factor inhibitor autoantibody is a rare condition that can be associated with pregnancy and the peripartum period. When it does occur during pregnancy, it most often occurs postpartum but can rarely appear before delivery. The patients present with a large sweep of bleeding manifestations that can pose diagnostic and management challenges. Elevated PTT that fails to correct by adding fresh plasma suggests the presence of the inhibitor and should trigger further workup. As cases are extremely rare, increased awareness and further studies are needed so that prompt intervention can be achieved.

## Figures and Tables

**Table 1:** Laboratory results.

Parameter	Patient	Reference range
PT	11.8 s	9.8–12.6 s
INR	1.05	0.87–1.15
PTT	92.1 s	24.3–36.7 s
Coagulation factor VIII activity	<1%	50–180%
Factor VIII inhibitor titer	74.9 BU	<0.6 BU
vWF Ag	190%	50–217%
vWF ristocetin cofactor	122%	42–200%
vWF Ag, multimeric	Normal	
PTT-La screen	80 s	<40 s
Hexagonal phase confirm	Negative	
DRVVT screen	22 s	<45 s
DNA ds Ab	0.9 IU/mL	<10 IU/mL
RNP 70	0.3 U/mL	<7.0 U/mL
Rheumatoid factor quantitative	44.6 IU/mL	<14.0 IU/mL
ANA	>32.0 ratio	>1.0 ratio
Cardiolipin Ab IgG	<14 GPL	< or = 14
Cardiolipin Ab IgM	<12 GPL	< or = 14
B2-glycoprotein IgM Ab	<9 GPL	< or = 20
B2-glycoprotein IgG Ab	<9 GPL	< or = 20
Anticentromere antibody	<0.4 U/mL	<7.0 U/mL
Jo 1 antibody	<0/3 U/mL	<7.0 U/mL
Scl 70 antibody	<0.6 U/mL	<7.0 U/mL
Smith antibody (Sm)	<0.8 U/mL	<7.0 U/mL
SS-A/Ro antibody	>240 U/mL	<7.0 U/mL
SS-B/La antibody	97 U/mL	<7.0 U/mL
U1RNP antibody	0.6 U/mL	<7.0 U/mL
Complement C3	174 mg/dL	87–200 mg/dL
Complement C4	20 mg/dL	19–52 mg/dL
Immunoglobulin A	242 mg/dL	66–443 mg/dL
Antistreptolysin O	<1 IU/mL	<250 IU/mL
ANCA	Negative	
Urine culture	Negative	
Random urine protein/creatinine ratio	858 mg/gm	161 mg/gm

**Table 2:** Review of the literature.

Article	Citation	Presentation	Findings	Outcome
41	Michiels et al. [4]	37 y/o G1P1 presented 4 months postpartum with bruises and hemarthrosis.	FVIII-0.02 U/mL FVIII inhibitor: 7.9 BU	Treated with prednisone taper of 1 mg/kg with remission within 2 months while on 20 mg/d without recurrence at 5-year f/u.
		22 y/o G3P3 presented 7 months postpartum with menorrhagia and bleeding after tooth extraction.	FVIII: 0.01–0.02 U/mL FVIII inhibitor: 12 BU	Treatment with cyclophosphamide 100 mg/day (0.7 mg/kg/d) without inhibitor disappearance; however, spontaneous remission developed 10 months later after discontinuing medication. Subsequent pregnancy without bleeding diathesis.
		31 y/o G2P2 presented 2 months postpartum with muscle and soft tissue bleeding, menorrhagia, ecchymoses, and hemarthrosis.	FVIII: <0.01 U/mL FVIII inhibitor: 24 BU	Treated with 4000 U human factor VIII, prednisone 1 mg/kg/d for 6 weeks, and cyclophosphamide 2 mg/kg/d concomitantly from the 3^rd^ to 6^th^ week of prednisone treatment, with a 5-day course of high-dose gamma-globulin at 0.5 g/kg/day. Recurrence of the inhibitor with removal of agents resulting in IVIG being initiated. Remission at 28 months.
		24 y/o G2P1A1 presented after delivery of stillborn with PPH and subsequent hemarthrosis.	FVIII: <0.01 FVIII inhibitor: 295–625 BU	Treated with 1 mg/kg prednisone for 3 weeks without remission. Five years later presented with FVIII 600 BU and thus 10K U of cryoprecipitate. At 24 years after treatment, the patient still had not achieved remission.

38	Coller et al. [5]	22 y/o primigravid woman presented with severe vaginal bleeding postpartum day 6.	PT: 12.6/13.0 sec PTT: 74/45 sec FVIII: 8% FIX: 90% FVIII inhibitor: 15 BU von Willebrand factor: 90%	Treated with 60 mg prednisone daily, became free of inhibitor within months, and had a successful second pregnancy without any subsequent bleeding diathesis.

6	Chaari et al. [9]	19 y/o F presented PPD one with large vaginal hematoma.	aPTT: 87/30 sec FVIII: 7% FVIII inhibitor: 64 BU	Treatment began with initial surgical intervention for evacuation of hematoma but was complicated by bleeding into the abdominal cavity and genitalia. The patient was given recombinant FVIIa and activated prothrombin complex concentrates. Due to symptom progression, the patient started on prednisone alone for 3 days followed by cotherapy with cyclophosphamide without symptom improvement. The patient passed away PPD 2/2 hemorrhagic shock.

5	Seethala et al. [7]	36 y/o woman presented with PPH s/p NSVD secondary to acquired factor VIII deficiency.	PTT: 71.7/45 sec PT: 15.9 sec INR: 1.3 FVIII: <1% FVIII inhibitor: >54.3 BU	Received factor VII and desmopressin. Treated with methylprednisolone, cytoxan, and plasmapheresis with appropriate decline in PTT but was discontinued because of sepsis prior to discharge. The patient was readmitted for bacteremia and succumbed to severe sepsis.

4	Kotani et al. [8]	31 y/o G1P1p developed postpartum hemorrhage after delivery. A female neonate developed subcutaneous hemorrhage on dorsum of hand on day 1of life s/p routine blood draw secondary to transplacental transfer of the inhibitor.	PT: 10.6 sec PTT: 76.9 sec FVIII: <1% FVIII inhibitor: 458 BU/mL PT: 14.3 sec PTT: 80.3 sec FVIII: <1% FVIII inhibitor: 199 BU/mL	Treatment for 31 y/o F consisted of 19 U PRBCS, 51 U FFP, steroid pulse therapy, and 3 vials of factor VII during hospital course and discharged on 30 mg/day of prednisolone. 10 months postpartum PTT WNL without any further complications. Neonate required no treatment and factor VIII, and PTT returned to baseline at postnatal month 4.
35	Chaari et al. [6]	31 y/o woman presented with several areas of ecchymoses over lower extremities several days prior to delivery.	FVIII: 18% FVIII inhibitor: 1, 4 BU	Treated with prednisone and rituximab.

8	Porteous et al. [10]	32 y/o primigravid woman developed postpartum hemorrhage hours after delivery.	PTT: 78.2 sec PT: 10.9 sec FVIII: <1% FVIII inhibitor: 15 BU	Treated with 1 mg/kg/day (60 mg) prednisolone, 5 doses of recombinant FVIIa 360 IU, 2 doses of DDAVP 0.3 *µ*g/kg, 20 U PRBCs, tranexamic acid 1 g daily over 20 days with continued bleeding. Bleeding was controlled with bilateral internal pudendal artery embolization. Maintenance therapy was achieved with prednisolone 60 mg and tranexamic acid 500 mg tds.

23	Sebastian et al. [11]	25 y/o F with a history of secondary APS in the setting of SLE and acquired factor VII inhibitor at 10 weeks of gestation developed retroperitoneal bleeding. Prior to current conception, the patient was symptomatic having suffered from several areas of ecchymoses on extremities, episcleritis, and urticaria and having been diagnosed with a PE 6 months prior.	PTT: 94/37 sec FVIII: 1.33% FVIII inhibitor: 614.4 BU C3: 0.57 C4: <0.08 Anti-ANA: 1 : 320 SS-A and Ro52: present Anti-dsDNA: 18.35/100 IU/mL	Treatment consisted of methylprednisolone IV, recombinant factor VII, prednisone 1 mg/kg, and cyclosporine 250 mg/day. The patient consented to terminate pregnancy. Continued maintenance therapy with cyclosporine 200 mg/day, chloroquine 250 mg/day, and methylprednisolone.

7	Rodrigues et al. [12]	34 y/o primigravid F presented PPD forty with complaints of spontaneous hematomas on extremities and intramuscular bleeding on the back, forearms, ankle, and thighs.	APTT: 62/<38 sec FVIII: 3.5% FVIII inhibitor: 10 BU FIX: 124% Fibrinogen: 430/450 mg/DL	Treated with prednisone 1 mg/kg/d plus tranexamic acid. The patient had remission of the disease with normalization of FVIII without the presence of the inhibitor in ∼2 years.

29	Lee et al. [13]	18 y/o F primigravid presented 9 months postpartum with painful swelling of knees and ankles, multiple bruises on hands and feet, and menorrhagia.	PT: 10.2/(10–14 sec) INR: 0.95/1.5 sec APTT: 84.3/40 sec FVIII: 1.4% Anti-FVIII: 28 BU	Treated with activated prothrombin complex concentrate and FEIBA anti-inhibitor coagulant complex at a dose of 50–100 units/kg q12 h. Corticosteroids were considered but not indicated due to remission of symptoms.

39	Azam et al. [14]	26-year-old multiparous woman presented PPD 2 with hemoperitoneum following lower segment C-section.	PTT: 90 sec (0–32 sec) Normal PT/INR FVIII level: 1% Positive inhibitor Normal lupus anticoagulant Normal vWF Normal antiphospholipid IgM and IgG	Treated with prednisone 60 mg/day initially with taper and initiation azathioprine.

2	Qian et al. [15]	35-year-old PPD 48 with chest pain and was found to have pleural hemorrhage.	PT: 15.2 sec PTT: 68.40 sec FVIII inhibitor: positive Inhibitor: 7.4%	Treated with aPCC, human factor VIII concentrates, corticosteroids, and plasma. At 6 months, no recurrence.
